# *In vitro* synergistic cytotoxicity of gemcitabine and pemetrexed and pharmacogenetic evaluation of response to gemcitabine in bladder cancer patients

**DOI:** 10.1038/sj.bjc.6603242

**Published:** 2006-07-25

**Authors:** V Mey, E Giovannetti, F De Braud, S Nannizzi, G Curigliano, F Verweij, O De Cobelli, S Pece, M Del Tacca, R Danesi

**Affiliations:** 1 Division of Pharmacology and Chemotherapy, Department of Internal Medicine, University of Pisa, 55, Via Roma, I-56100, 56126, Pisa, Italy; 2 Department of Medical Oncology and Division of Urology, European Institute of Oncology, I-20141 Milan, Italy

**Keywords:** bladder cancer, antifolates, gemcitabine, drug combination, inducible gene expression

## Abstract

The present study was performed to investigate the capability of gemcitabine and pemetrexed to synergistically interact with respect to cytotoxicity and apoptosis in T24 and J82 bladder cancer cells, and to establish a correlation between drug activity and gene expression of selected genes in tumour samples. The interaction between gemcitabine and pemetrexed was synergistic; indeed, pemetrexed favoured gemcitabine cytotoxicity by increasing cellular population in S-phase, reducing Akt phosphorylation as well as by inducing the expression of a major gemcitabine uptake system, the human equilibrative nucleoside transporter-1 (hENT1), and the key activating enzyme deoxycytidine kinase (dCK) in both cell lines. Bladder tumour specimens showed an heterogeneous gene expression pattern and patients with higher levels of dCK and hENT1 had better response. Moreover, human nucleoside concentrative transporter-1 was detectable only in 3/12 patients, two of whom presented a complete response to gemcitabine. These data provide evidence that the chemotherapeutic activity of the combination of gemcitabine and pemetrexed is synergistic against bladder cancer cells *in vitro* and that the assessment of the expression of genes involved in gemcitabine uptake and activation might be a possible determinant of bladder cancer response and may represent a new tool for treatment optimization.

Bladder cancer is the fourth cause of death from cancer in the Western world and its incidence and mortality rates have risen steadily over the past decade (Parkin *et al*, 1999; Jemal *et al*, 2005).

Transitional cell carcinoma (TCC) comprises more than 90% of all bladder cancers, and superficial tumours (Ta–T1 stages) account for 70–80% of newly diagnosed TCC. However, despite the macroscopically complete eradication of the primary lesion, approximately two-thirds of patients will recur, with a worsening of tumour grade and stage (Scher *et al*, 1997). The most effective approach against superficial bladder cancer is intravesical immunotherapy with Calmette-Guerin *Bacillus*, but it is associated with serious morbidity and it does not result in a significant survival improvement (Crawford, 2002). Similarly, systemic combination chemotherapy, such as the methotrexate, vinblastine, doxorubicin and cisplatin (MVAC) regimen, has proven activity in advanced bladder cancer, but it exhibits a significant toxicity burden, with a treatment-related mortality of about 4% (Chester *et al*, 2004). Therefore, a great deal of interest has been focused on research into new drugs or new drug combinations for intravesical and systemic chemotherapy. In particular, gemcitabine proved to be active both by intravescical instillation treatment, with minimal bladder irritation and systemic administration (Moore *et al*, 1997; Laufer *et al*, 2003). Indeed, the gemcitabine-cisplatin combination is effective and safe and it is now frequently administered as first-line therapy against metastatic bladder cancer (von der Maase *et al*, 2000), although, a significant proportion of patients are not eligible to receive cisplatin chemotherapy (Chester *et al*, 2004; Li *et al*, 2005).

Gemcitabine, a cytotoxic pyrimidine deoxynucleoside analogue, is transported into the cell mostly by human equilibrative and concentrative nucleoside transporters (hENT and hCNT, respectively). Cells deficient in hENT1 are highly resistant to gemcitabine (Mackey *et al*, 2003) while hCNT1 transfection increases gemcitabine sensitivity in pancreatic cancer cell lines (Garcia-Manteiga *et al*, 2003). As a prodrug, gemcitabine must be phosphorylated to its active diphosphate and triphosphate metabolites, which inhibit ribonucleotide reductase (RR) and DNA synthesis, respectively. Deoxycytidine kinase (dCK) is the rate-limiting enzyme in the biotransformation of nucleoside analogs and the increase in dCK activity may improve the efficacy of gemcitabine (Blackstock *et al*, 2001). Moreover, dCK activity was related to dCK mRNA levels in bladder cancer specimens as well as in tumour xenografts, and preliminary data in esophageal tumours demonstrated a significant correlation between dCK expression and response to gemcitabine-based treatment (Sigmond *et al*, 2004). Furthermore, high expression of the catabolic enzymes 5’-nucleotidase (5’-NT) and cytidine deaminase (CDA) has been found in many cell lines resistant to gemcitabine (Eda *et al*, 1998; Lotfi *et al*, 2001). Finally, non-small cell lung cancer patients with low expression of the M1 subunit of RR (RRM1) significantly benefited from gemcitabine/cisplatin neoadjuvant chemotherapy (Rosell *et al*, 2004), while resistance to gemcitabine was observed both in RRM1 and RRM2 overexpressing cells (Goan *et al*, 1999; Davidson *et al*, 2004).

Modulation of enzymes involved in gemcitabine uptake and metabolism may influence drug activity against human tumour cell lines and, among chemotherapeutic drugs, pemetrexed appears to be a potential candidate because of its ability to deplete cellular nucleotide pools by inhibiting nucleotide synthesis (Giovannetti *et al*, 2005). Indeed, pemetrexed and its polyglutamates are potent, tight-binding inhibitors of folate-dependent enzymes, including thymidylate synthase (TS), dihydrofolate reductase (DHFR), and glycinamide ribonucleotide formyltransferase (GARFT) and show activity against a wide variety of solid tumours, including bladder cancer (Calvert, 2004). This new multitargeted antifolate may enhance the expression of hENT1 and dCK as a compensatory mechanism, potentially favouring gemcitabine activity against cancer cells. Thus, the primary objective of this study was the analysis of cellular and genetic aspects underlying the pharmacological interaction of gemcitabine and pemetrexed in two human bladder cancer cells. Moreover, this study was aimed at providing a preliminary characterisation of the expression pattern of hENT1, hCNT1, dCK, 5’-NT, CDA, RRM1 and RRM2 in surgical specimens of bladder cancer, in order to find a possible association between gene expression and response to gemcitabine treatment.

## MATERIALS AND METHODS

### *In vitro* studies

#### Drugs and chemicals

Gemcitabine (difluorodeoxycytidine, dFdC) and pemetrexed (multitargeted antifolate, MTA) were generous gifts of Eli Lilly (Indianapolis, IN, USA). Drugs were dissolved in sterile distilled water and diluted in culture medium immediately before use. McCoy's medium, fetal bovine serum (FBS), L-glutamine, penicillin and streptomycin were from Gibco (Gaithersburg, MD, USA). All other chemicals were from Sigma Chemical Co. (St Louis, MO, USA).

#### Cell lines

Human bladder TCC cells T24 and J82 were obtained from American Type Culture Collection (Manassas, VA, USA). Cells were maintained as monolayer cultures in McCoy's (T24) and MEM Eagle (J82) medium, supplemented with 10% fetal bovine serum, L-glutamine (2 mM), penicillin (50 IU ml^−1^) and streptomycin (50 *µ*g ml^−1^). Cells were cultivated in 75 cm^2^ tissue culture flasks (Costar, Cambridge, MA, USA), at 37°C in 5% CO_2_ and 95% air, and harvested with trypsin-EDTA when they were in logarithmic growth.

### Assay of cytotoxicity

Cytotoxicity was assessed by the CellTiter 96 Non-radioactive cell proliferation kit (Promega, Madison, MA, USA) based on the cellular metabolism of the tetrazolium compound MTT. Cells (5 × 10^4^ well^−1^) were seeded in 1 ml of medium in a 12-well plate and allowed to attach for 24 h. Cells were treated with: (1) gemcitabine (0.3 nM–33.3 *µ*M) for 1, 6, 24 and 48 h; (2) pemetrexed (2.1 nM–212.1 *µ*M) for 1, 6, 24 and 48 h; (3) gemcitabine for 1 h, followed by a 24-h washout in drug-free medium, and then pemetrexed for 24 h; (4) pemetrexed for 24 h, followed by a 24-h washout in drug-free medium, and then gemcitabine for 1 h. At the end of drug exposure, sample processing was performed as indicated by the manufacturer. Cell growth inhibition was expressed as the percentage of the 570 nm absorbance relative to untreated control cultures, measured with a microplate reader (Multiskan Spectrum, Vantaa, Finland), and the 50% inhibitory concentration of cell growth (IC_50_) was calculated by sigmoid inhibition model (GraphPad PRISM *version* 4.0; Intuitive Software for Science, San Diego, CA, USA).

Drug interaction was assessed at a fixed 1 : 1 concentration ratio of gemcitabine-pemetrexed by using the combination index (CI) of Chou *et al* (1994), where CI<1, CI=1 and CI>1 indicated synergistic, additive and antagonistic effects, respectively. Data analysis was performed by the Calcusyn software (Biosoft, Oxford, UK).

### Cell cycle analysis and apoptosis

Cells (10^6^ well^−1^) were plated in 10 ml in 100-mm Petri dishes (Costar) and allowed to grow for 24 h. Cells were treated with gemcitabine (1 h), pemetrexed (24 h), and their combinations at concentrations corresponding to IC_50_ levels and were harvested immediately after the end of drug exposure or allowed to grow for additional 24 h in drug-free medium. Then cells were washed twice with PBS and DNA was stained with a solution containing propidium iodide (25 *µ*g ml^−1^), RNase (1 mg ml^−1^) and Nonidet-P40 (0.1%). Samples were kept on ice for 30 min and cytofluorimetry was performed using a FACScan (Becton Dickinson, San José, CA, USA). Data analysis were carried out with the CELLQuest (Becton Dickinson) and Modfit softwares (Verity Software, Topsham, ME, USA).

Apoptosis was evaluated in cells treated with gemcitabine, pemetrexed and their combinations at IC_50_ levels, as described in ‘Assay of Cytotoxicity’. At the end of incubation, cells were washed twice with PBS and fixed in 4% buffered paraformaldehyde for 15 min. Cells were resuspended and incubated for further 15 min in a solution containing 8 *µ*g ml^−1^ bisbenzimide chloride. Cells were spotted on glass slides and examined by fluorescence microscopy (Leica, Berlin, Germany). A total of 200 cells from randomly chosen microscopic fields were counted and the percentage of cells displaying chromatin condensation and nuclear fragmentation relative to the total number of counted cells (apoptotic index) was calculated.

### Assay of Akt phosphorylation

Akt protein phosphorylation after gemcitabine or pemetrexed treatment, described above for cell cycle analysis, was assayed with a P-Ser473 specific ELISA and normalised to the total Akt content following the manufacturer's instructions (BioSource International, Camarillo, CA, USA). P-Ser473 Akt and Akt total concentrations were calculated from standard curves and values of P-Ser473 Akt were normalised for total Akt and protein content, which was measured with the Lowry reagent (Sigma), as described previously (Giovannetti *et al*, 2005).

### Quantitative PCR analysis of cell lines

Cells were treated with gemcitabine and pemetrexed at IC_50_ and IC_10_ concentration values. RNA was extracted with the QiaAmp RNA mini Kit (Qiagen, San Diego, CA, USA) according to the manufacturer's protocol, dissolved in 10 mM dithiothreitol and 200 U ml^−1^ RNase inhibitor in Rnase-free water, and measured by absorbance reading at 260/280 nm, using the Uvikon-940 spectrophotometer (Kontron, Milan, Italy). RNA (1 *µ*g) was reverse transcribed at 37°C for 1 h in 50-*µ*l reaction volume containing 0.8 mM dNTPs, 200 U of MMLV-RT, 40 U of RNase inhibitor and 0.05 *µ*g ml^−1^ of random primers. The resulting cDNA was amplified by quantitative PCR with the Applied Biosystems 7900HT sequence detection system (Applied Biosystems, Foster City, CA, USA). PCR reactions were performed in triplicate using 5 *µ*l of cDNA, 12.5 *µ*l of TaqMan Universal PCR Master Mix, 2.5 *µ*l of probe and 2.5 *µ*l of forward and reverse primers in a final volume of 25 *µ*l. Samples were amplified using an initial incubation at 50°C for 5 min, followed by incubation at 95°C for 10 min, 40 cycles of denaturation at 95°C for 15 s followed by annealing and extension at 60° for 1 min.

Forward and reverse primers and probes for dCK (NM_000788), cN-II 5′-NT (NM_012229), CDA (NM_001785), TS (NM_0010711), DHFR (NM_000791) and GARFT (NM_000819) were designed on the basis of the gene sequence obtained from the GeneBank database with the Primer Express software (Applied Biosystems, V.2.0), as previously described (Giovannetti *et al*, 2005); while primers and probes for hENT1 (NM_004955), hCNT1 (NM_004213) and for the regulatory M1 (NM_001033), and catalytic M2 (NM_001034) subunits of RR were obtained from Applied Biosystems Assay-on-Demand^®^ products (Hs00168784, Hs00188418, Hs0035724 and Hs00191940).

Amplifications were normalised to glyceraldehyde 3-phosphate dehydrogenase (GAPDH), and quantitation of gene expression in treated cells was performed using the ΔΔ*C*_T_ calculation, where *C*_T_ is the threshold cycle. The amount of target gene, normalised to GAPDH and relative to the calibrator (untreated control cells), was reported as percent variation of 2^−ΔΔ*C*_T_^ with respect to control.

### Modulation of gemcitabine uptake, metabolism and cytotoxicity

Cells plated and treated with gemcitabine alone or in combination with pemetrexed (0.3 nM–33.3 *µ*M) as described in ‘Assay of Cytotoxicity’, were simultaneously exposed to 2′-deoxycytidine (10 *µ*M), to inhibit drug activation by phosphorylation (dCK), to the specific hENT1 inhibitor nitrobenzylthioinosine (NBMPR, 100 nM) or to dipyridamole, a non-specific inhibitor of nucleoside transporters, used at concentration of 10 *µ*M, as previously described (Giovannetti *et al*, 2005).

### *Ex vivo* studies on human samples of bladder cancer

#### Patient characteristics and treatment

In all, 12 bladder cancer patients were evaluated in this study. Median age was 61 years (range, 32–75); five were males and seven females. Six patients (50.0%) had stage I, while 16.7% had stage II and 33.3% stage III disease at the time of diagnosis.

Chemotherapy treatment consisted of intravesical gemcitabine administered every 7±1 day for 6 consecutive weeks. Gemcitabine was reconstituted in 0.9% NaCl solution for injection, to a concentration of 40 mg ml^−1^ and 2000 mg were delivered intravescically, through a urethral catheter, which was then removed. The patient was asked to avoid urination for 1 h after gemcitabine instillation.

#### Tissue sampling and RNA extraction

The experimental protocol was approved by the local Ethics Committee and patients were required to sign a consent form to use pathological specimens for research purposes, prior to their enrolment.

Tissue sampling was performed immediately after transurethral resection, 7 days before the first instillation of gemcitabine. Tumours were fragmented, placed in optimal cutting temperature (OCT) solution (Sakura Finetek Inc., Torrance, CA, USA), and stored at −80°C until extraction of RNA by the TRI REAGENT LS (Sigma) from tumour fragments homogenised at 4°C. RNA concentration was determined by absorbance reading at 260 nm.

#### Quantitative PCR analysis in tissue samples

RNA extracted from tissue specimens was reverse transcribed in a 50 *µ*l reaction volume and the resulting cDNA was amplified by quantitative, real-time PCR, as described above in ‘Quantitative PCR analysis of cell lines’. Preliminary experiments were carried out with dilutions of cDNA obtained from Quantitative PCR Human Reference Total RNA (Stratagene, La Jolla, CA, USA) to determine the primer concentrations that give the minimum s.d. among *C*_T_ values and to demonstrate that the efficiencies of amplification of targets and reference genes (GAPDH) were similar. All reactions were performed in triplicate, with appropriate nontemplate controls, and the coefficient of variation (CV) was <1% for all replicates.

### Statistical methods

All experiments were performed in triplicate and data were expressed as mean values±s.d. and were analysed by Student's *t*-test or ANOVA, followed by the Tukey's test for multiple comparisons. Demographic and clinical informations were obtained from medical records. Patients were endoscopically evaluated for response within 1–4 weeks after the end of treatment, using a marker lesion, as previously described (Lamm *et al*, 1995). Indeed, trials conducted within the EORTC GU and MRC Group using marker tumours provided evidence that this approach is safe and ethically acceptable (Van der Meijden *et al*, 1996).

The relationship between expression of target genes and response to treatment was evaluated by stratifying patients by clinical outcome and gene expression values. Data were analysed using SPSS/PC+11.5 statistical software (LEAD Technologies, NC, USA) and statistical significance was set at *P*<0.05.

## RESULTS

### Cytotoxicity and pharmacological interaction

**Figure 1:**
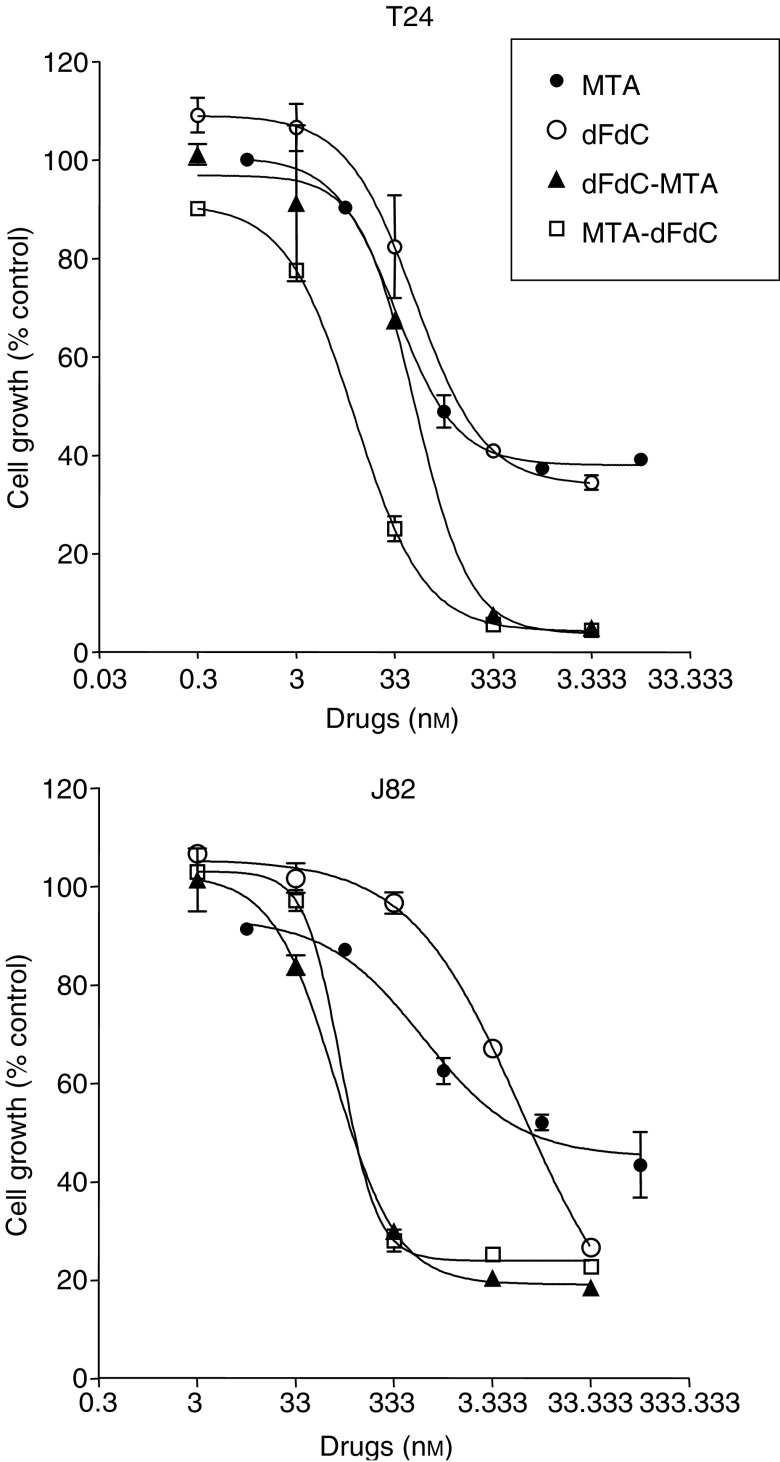
Concentration-dependent cytotoxicity of gemcitabine (dFdC), pemetrexed (MTA), and their combinations in T24 and J82 bladder cancer cells. Each data point represents the percentage of proliferating cells with respect to untreated control and is the average of three independent experiments. *Bars*, s.d.

Gemcitabine was cytotoxic against T24 and J82 bladder cancer cells, with IC_50_s of 91.7±5.1 and 4366.7±833.3 nM, after 1 h treatment, and 6.1±0.4 and 5.7±1.0 nM after 24 h exposure, respectively (Figure 1 and Table 1). A dose-dependent inhibition of cell growth was also observed in both cell lines after 24 and 48-h pemetrexed exposures, with a higher sensitivity of T24 than J82 cells.

**Figure 2:**
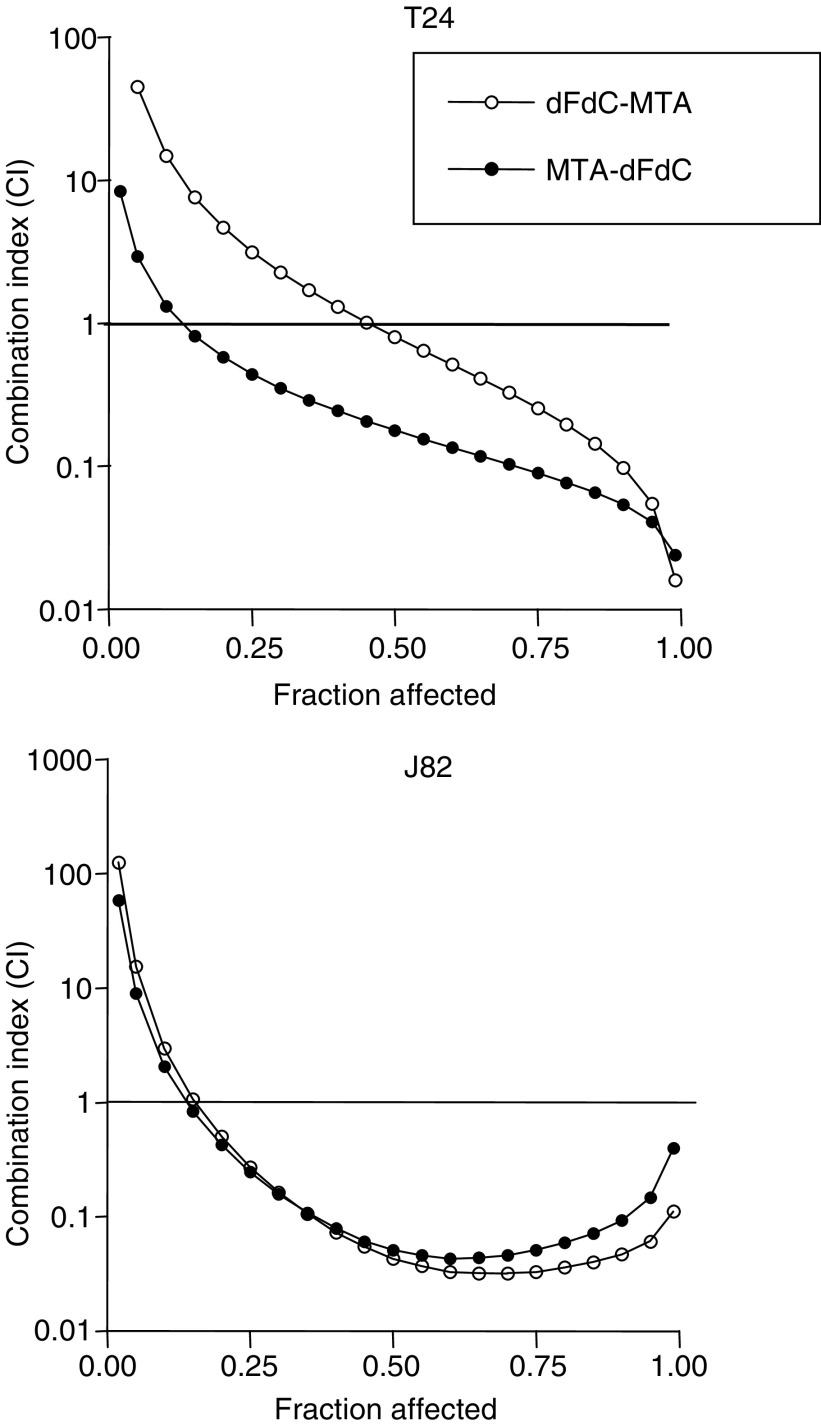
Isobologram analysis of pharmacologic interaction of gemcitabine (dFdC) – pemetrexed (MTA) combinations in T24 and J82 cells.

As the CI method recommends a ratio of IC_50_s values at which drugs are equipotent, combination studies were performed at fixed 1 : 1 (gemcitabine:pemetrexed) concentration ratios in both bladder cancer cells. The sequential exposure of cell lines to pemetrexed followed by gemcitabine reduced the IC_50_s of gemcitabine to 10.7±0.7 and 100.0±7.3 nM, in T24 and J82 cells, respectively, while the IC_50_s resulting from the reverse sequence were 47.3±3.7 and 83.3±9.7 nM (Figure 1). The analysis of drug interaction revealed a strong synergistic (CI<0.3) at mid- to high fractions effects using both schedules in T24 and J82 cells, whereas CI values at fractional effects of 0.25 were close to 1, indicating moderate synergism or additivity, and CI values at frational effects of 0.10 displayed some antagonistic effects. The sequence pemetrexed → gemcitabine proved to be the most effective at all drug concentrations in T24 cells, while both sequences were almost equivalent on J82 cells (Figure 2).

### Cell cycle activity

Pemetrexed was able to affect the cell cycle of bladder cancer cells (Table 2). In particular, the percentage of T24 and J82 cells in the S-phase increased significantly (*P*<0.05) after treatment with pemetrexed for 24 h. The same effect on T24 cell cycle was observed after a 1-h treatment with gemcitabine. In contrast flow cytometry did not show significant perturbation after gemcitabine exposure in J82 cells (Table 2). Furthermore, drug combinations demonstrated that both schedules increased the percentage of cells in the S-phase, while in the J82 cells there was a similar reduction in the G1 phase (Table 2).

### Induction of apoptosis

**Figure 3:**
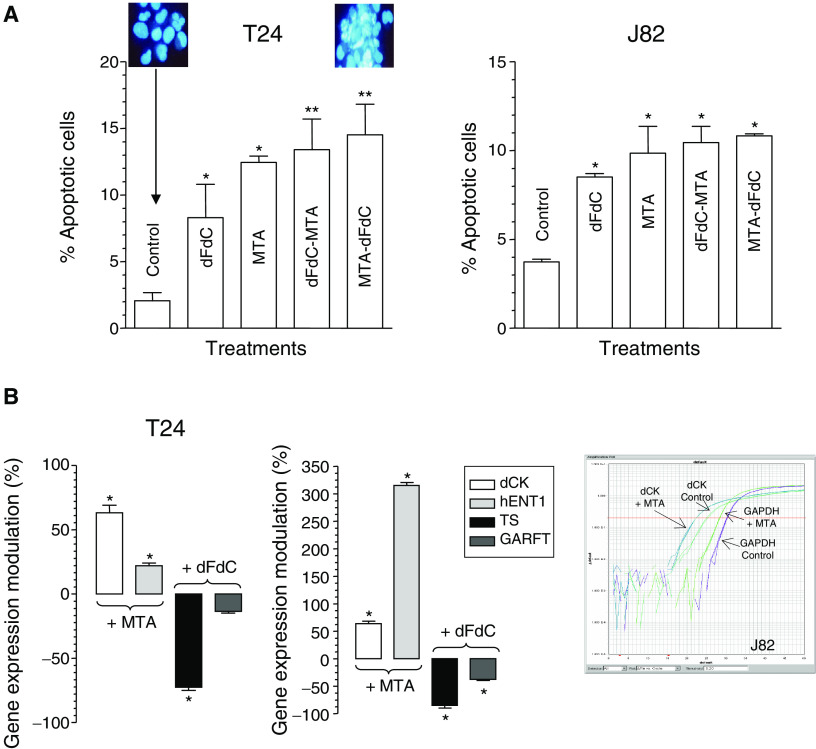
Percentage of cells with damaged DNA after drug treatments with gemcitabine (dFdC), pemetrexed (MTA) and their combinations in both cell lines (**A**). *Upper panels*, morphological appearance of control and treated cells. Modulation of hENT1 and dCK expression by pemetrexed and modulation of TS and GARFT expression by gemcitabine in comparison with control in T24 and J82 cells (**B**). *Right panel*, representative plot of dCK and GAPDH expression in control and treated J82 bladder cancer cells. Columns, mean values obtained from three independent experiments; *bars*, s.d. ^*^P<0.05 with respect to control, ^**^P<0.05 with respect to gemcitabine.

Bladder cancer T24 and J82 cells exposed to pemetrexed, gemcitabine and their combinations presented typical apoptotic morphology with cell shrinkage, nuclear condensation and fragmentation, and rupture of cells into debris. Furthermore, the occurence of apoptosis was significantly higher in pemetrexed-treated cells *vs* controls, whereas gemcitabine exposure was associated with a lower percentage of apoptotic cells. In each case, the drug combinations significantly increased the apoptotic index of bladder cancer cells with respect to controls (Figure 3). Finally, both drug combinations significantly increased the apoptotic index of T24 cells with respect to gemcitabine-treated cells (*P*<0.05).

### Inhibition of Akt phosphorilation

**Figure 4:**
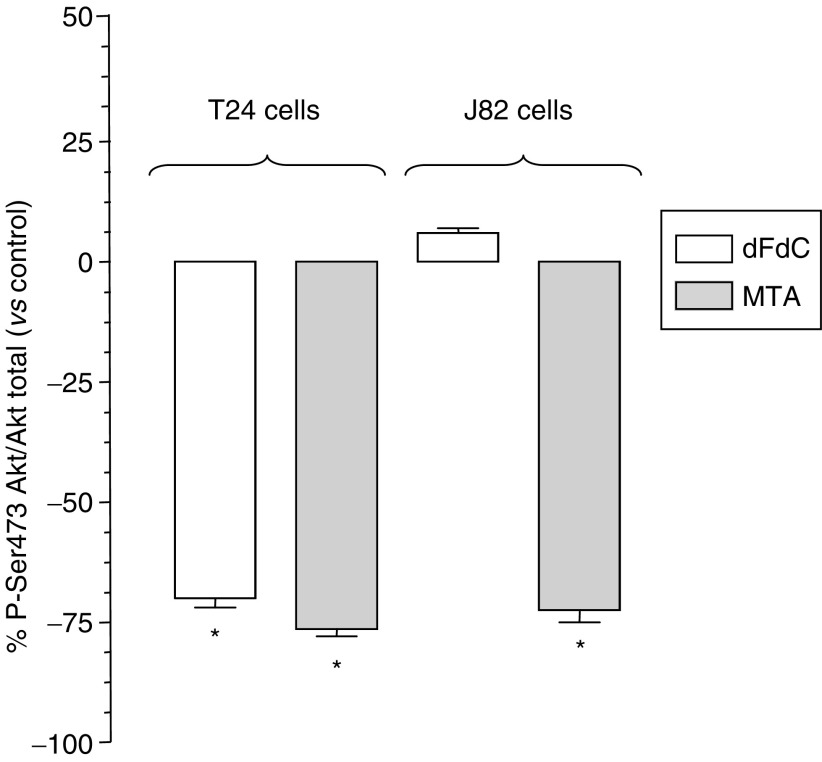
Reduction of P-Ser473 Akt by gemcitabine (dFdC) and pemetrexed (MTA) in bladder cancer cell lines T24 and J82. ^*^P<0.05 with respect to control.

Pemetrexed and gemcitabine significantly reduced the amount of phosphorylated Akt in T24 cells. In J82 cells, the amount of the phosphorylated Akt was decreased up to −73.9% by pemetrexed (*P*<0.05), while gemcitabine was unable to modulate the activation of Akt (Figure 4).

### Gene expression and chemosensitivity

The relative expression of dCK and hENT1 was higher in T24 and J82 cells (dCK, 0.89 *vs* 0.82 and hENT1, 0.88 *vs* 0.85). Therefore, the lower chemosensitivity of J82 cells with respect to T24 cells appeared likely dependent on lower expression of genes encoding for hENT1 and dCK, which are involved in drug transport and activation, respectively. A similar correlation was found between the IC_50_ values of pemetrexed and the target enzyme TS, its relative expression being higher (J82) as compared to T24 cells (TS, 1.02 *vs* 0.83).

### Modulation of dCK, hENT1, TS and GARFT gene expression

Pemetrexed significantly increased hENT1 expression in both cell lines (*P*<0.05). In particular, a 24-h pemetrexed exposure at its IC_50_s levels resulted in a three-fold increase in hENT1 expression in J82 cells (Figure 3). Similar results were observed for dCK, which expression was increased by pemetrexed up to 57.7% (T24 cells) and 68.2% (J82 cells), respectively (Figure 3) while at IC_10_ pemetrexed levels here was only a minimal enhancement of hENT1 expression in J82 cells. Moreover, gemcitabine exposure modulated TS and GARFT expression in both cell lines. In particular, TS expression was significantly decreased up to −81.7 and −74.0% in J82 and T24 cells, respectively. Similar results on GARFT were observed at gemcitabine concentration corresponding to its IC_50_, GARFT expression being reduced up to −36.04% in J82 cells, while no significant changes were observed in T24 cells (Figure 3).

### Effect of inhibition of gemcitabine metabolism and transport on cytotoxicity

A key role for dCK and hENT1 on sensitivity of bladder cell lines to gemcitabine was demonstrated by the increase in IC_50_ values after simultaneous treatment with their inhibitors (Table 3). In particular, incubation with both dipyridamole and NBMPR resulted in up to 49-fold increase in IC_50_s, suggesting a pivotal role for hENT1 in gemcitabine uptake. In contrast, the use of sequential administration of gemcitabine and pemetrexed partially prevented the reduction of cytotoxic activity by simultaneous administration of deoxycytidine in both cell lines. Similar results were obtained for both schedules with dipyridamole and NBMPR in T24 cells. However, the use of the drug combinations, totally protected J82 cells from the reduction of antiproliferative effect caused by the inhibition of hENT1 (Table 3).

### Clinical outcome and response to chemotherapy

Clinical data are available from 12 patients with bladder TCC, whose tumour specimens underwent a pathologic examination and were stored in the tissue bank. Patients’ follow-up ranged from 5 to 16 months (median 10.5 months) after surgery. All patients received gemcitabine, as described above, and were evaluable for response; two complete responses (16.7%) and 10 stable diseases (83.3%) were observed.

### Gemcitabine-related gene expression levels in patients

**Figure 5:**
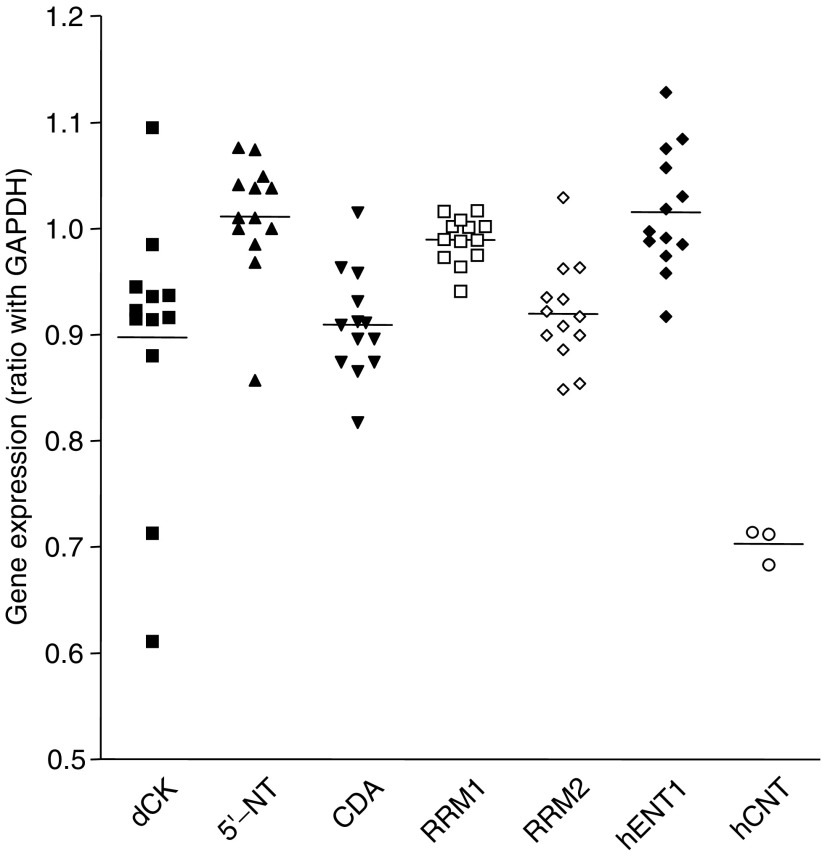
Gene expression of gemcitabine-related genes in 12 bladder cancer patients. Values of gene expression were calculated by the GAPDH/target gene ratio in triplicate experiments.

The plot on Figure 5 shows the variability of gene expression observed in patients. The results of gene expression analysis of tissue samples showed that dCK, 5′-NT, CDA, RRM1, RRM2 and hENT1 mRNAs were detectable in all samples, while hCNT1 mRNA was detectable in only three patients. The gene expression profile of patients showed a variable pattern. In particular, dCK was the gene displaying the most pronounced variability (from 0.611 to 1.095), while the variability of hENT1 (1.094±0.102), 5′-NT (1.010±0.053), CDA (0.911±0.058), RRM1 (0.988±0.023), and RRM2 (0.924±0.051), was moderate, suggesting a possible stratification of patients on the basis of their expression profile to create homogeneous groups with different likelihood to respond to gemcitabine treatment. Finally, no apparent relationships among the expression of different genes within individual patients were observed.

### Association between clinical outcome and gene expression levels

A significant relationship was observed between clinical response and expression levels of selected genes (Table 4). In particular, mean values of dCK and hENT1 were significantly higher in patients who had a complete pathological response. Furthermore, hCNT1 expression was detectable only in three patients of whom two presented complete response. However, the other patient with detectable hCNT1 expression presented the lowest level of dCK expression (0.611±0.016).

## DISCUSSION

Despite the recent advances in the use of chemotherapy in both local and metastatic bladder cancer, long-term, disease-free survival rates remain disappointing. During the past decade, gemcitabine gained widespread use for the treatment of bladder cancer (Moore *et al*, 1997; Fechner *et al*, 2003; Muramaki *et al*, 2004). Moreover, gemcitabine has shown single-agent response rates of 28–36% in previously untreated metastatic bladder patients, with mild myelosuppression (Chester *et al*, 2004). The clinical activity of gemcitabine observed in the present work by the data on complete response and stable disease rates, which were 16.7 and 83.3%, respectively, is in agreement with published data.

Nevertheless, there is a continuing need to develop more effective cytotoxic chemotherapy regimens, searching for agents with activity against bladder cancer and low toxicity, as well as to identify molecular markers that are predictive of response, in order to select chemotherapeutic agents best suited for the individual treatment of patients.

Among new drugs, pemetrexed has demonstrated its activity against a variety of tumour types, including bladder cancer, and it is generally well tolerated (Fechner *et al*, 2003).

In preclinical studies, the combination of pemetrexed and gemcitabine yielded conflicting results. Studies on colorectal cancer cell lines HCT-8, LoVo, WiDr, LRWZ and Calu-1 lung cancer cells showed maximal synergistic cytotoxicity when gemcitabine was followed by pemetrexed in cells (Tesei *et al*, 2002; Adjei, 2002; Giovannetti *et al*, 2005). On the contrary, other studies demonstrated that the schedule-dependent synergism was maximal when pemetrexed preceded gemcitabine in HT29 colon cancer cells (Tonkinson *et al*, 1999), MIA PaCa-2, PANC-1 and Capan-1 pancreatic cancer cells (Giovannetti *et al*, 2004) and A549 and Calu-6 lung cancer cells (Giovannetti *et al*, 2005). *In vitro* experimental data obtained in the present study indicate that in bladder cancer T24 cells the highest chemotherapeutic synergism was observed with the sequence pemetrexed → gemcitabine, while both sequences were almost equivalent in J82 cells.

Understanding the role of cell cycle, apoptosis or other mechanisms involved in cell death or proliferation, may be crucial to improve the therapeutic activity of anticancer drug combinations. The synergistic interaction reported in the present study may be attributed, at least in part, to cell cycle perturbations, inhibition of Akt phosphorilation and modulation of gene expression. Indeed, cell cycle evaluation demonstrated a shift towards the S-phase after pemetrexed exposure, potentially facilitating gemcitabine activity, and gemcitabine-pemetrexed combinations enhanced apoptosis. These findings are in agreement with previous data obtained in colon and pancreatic cells, which were synchronised after 24-h pemetrexed exposure, and showed a significant increase of apoptosis after treatment with gemcitabine-pemetrexed combinations (Tonkinson *et al*, 1999; Giovannetti *et al*, 2004).

One potential antiapoptotic signal transduction system that has been linked to chemoresistance of human cancer cells is the PI3K-Akt pathway. In particular, the reduction of phosphorylated Akt correlated with the enhancement of gemcitabine-induced apoptosis and antitumour activity, suggesting that the PI3K-Akt pathway plays a significant role in mediating drug resistance in several pancreatic cancer cells (Bondar *et al*, 2002). Moreover, an immunohistochemical analysis in patients who underwent curative resection revealed that p-Akt expression was a prognostic factor for overall survival in primary pancreatic adenocarcinoma (Yamamoto *et al*, 2004). Therefore, the inhibition of Akt might be a possible molecular target for novel therapeutic strategies and the present study demonstrated that pemetrexed significantly decreased the amount of the activated form of Akt.

The present *in vitro* experimental findings also suggest that the enhancement of hENT1 and dCK expression by pemetrexed could be responsible, at least in part, for the synergistic interaction obtained particularly at mid- to high frational effects with the sequential exposure to gemcitabine in both T24 and J82 bladder cell lines. Indeed, hENT1 and dCK gene expression was not significantly modulated in bladder cancer cells at low fraction affected (0.10 effect level), potentially explaining drug antagonism. However, the modulation of TS and GARFT expression by gemcitabine, potentially favouring pemetrexed activity, could explain the synergistic interaction observed with the gemcitabine → pemetrexed combination, especially in J82 cells. These results underline the importance of integrating gene expression analysis for rational development of cytotoxic drug combinations.

Similar results were reported in non-small cell lung cancer cells (Giovannetti *et al*, 2005), suggesting that modulation of dCK and hENT1 gene expression as well as inhibition of Akt phosphorylation by pemetrexed may be involved in the improvement of gemcitabine therapeutic potential against several cell lines.

Moreover, a recent study by Achiwa *et al* (2004) demonstrated that increased hENT1 expression was a determinant of gemcitabine sensitivity, while the decreased dCK expression was associated with acquired resistance to gemcitabine in lung cancer cells (Achiwa *et al*, 2004).

Finally, although the results should be considered very preliminary and the possible prognostic value of other determinants, such as other intracellular 5′-NT, clearly requires to be evaluated in detail, the present study provides the first evidence of a significant correlation between gemcitabine chemotherapy outcome and hENT1, and dCK expression, in bladder cancer specimens.

These findings are in agreement with a previous study on tissues from patients with advanced pancreatic cancer. Patients with detectable hENT1 expression had significantly longer median survival from gemcitabine initiation than those lacking hENT1 in a proportion of adenocarcinoma cells (median survival, 13 *vs* 4 months, *P*=0.01) (Spratlin *et al*, 2004).

Similar results were obtained in a pharmacogenetic study on 83 pancreatic cancer patients where PCR analysis demonstrated that overall survival was significantly longer in patients with high hENT1 expression, with respect to patients with low hENT1 levels (median, 25.7, 95% CI, 17.6–33.7 *vs* 8.5, 95% CI, 7.0–9.9 month) and the multivariate analysis confirmed the prognostic significance of hENT1 (Giovannetti *et al*, 2006).

In addition, several *in vitro* studies demonstrated that several cancer cell lines incorporate gemcitabine mostly via the hENT1 and hCNT1 transporters and that treatment of cells with nucleoside transport inhibitors NBMPR or dipyridamole markedly reduced the sensitivity to gemcitabine (Mackey *et al*, 2003).

The crucial role of hENT1 was confirmed in the present work by the marked reduction of gemcitabine activity with the nucleoside transport inhibitors which was partially circumvented after exposure to drug combinations, confirming the ability of pemetrexed to increase hENT1 expression. Furthermore, transcription analysis in untreated cells suggested the predictive value of expression of both hENT1 and dCK, while, as previously reported in lung and colon cancer cells, a similar correlation was found between TS and chemoresistance to pemetrexed (Sigmond *et al*, 2003; Giovannetti *et al*, 2005). Indeed, preclinical studies have also shown that pretreatment dCK expression level could be used as a predictive parameter of tumour sensitivity and a clear correlation between dCK activity and gemcitabine sensitivity was observed in several tumour xenografts (Kroep *et al*, 2002). Moreover, dCK mRNA expression in leukaemic blasts at diagnosis was correlated with clinical outcome in patients with acute myeloid leukaemia treated with cytarabine (Galmarini *et al*, 2003).

In light of these findings, we conclude that (1) gemcitabine and pemetrexed synergistically interact against bladder cancer cells, through suppression of Akt phosphorylation and induction of apoptosis; (2) pemetrexed enhances dCK and hENT1 expression in both cell lines thus suggesting that the sequence pemetrexedgemcitabine is mostly rationally designed; (3) the transcription analysis of gemcitabine-related genes in bladder cancer specimens is feasible and might be useful to help select patients with the highest likelyhood to enjoy complete response after gemcitabine therapy.

## Figures and Tables

**Table 1:** Cytotoxic effect and pharmacological interaction of gemcitabine and pemetrexed against bladder cancer cell lines

	**IC_50_ values (nM)^a^**			
	**T24**		**J82**	
**Times (h)**	**Gemcitabine**	**Pemetrexed**	**Gemcitabine**	**Pemetrexed**
1	91.7±5.1	6392.9±958.9	4366.7±833.3	38194.0±7638.8
6	35.2±2.9	156.6±26.6	166.7±33.3	25642.2±5384.8
24	6.1±0.4	64.9±5.3	5.7±1.0	2566.9±339.4
48	2.1±0.4	22.5±2.8	5.2±0.8	63.6±4.2
				

	**CI values^b^**			
**Fa**	**Gemcitabine-Pemetrexed**	**Pemetrexed-Gemcitabine**	**Gemcitabine-Pemetrexed**	**Pemetrexed-Gemcitabine**
0.50	0.80	0.17	0.04	0.05
0.75	0.25	0.09	0.03	0.05
0.90	0.09	0.05	0.05	0.09

a IC_50_s were calculated as mean values±s.d. of at least three MTT independent experiments after 2, 6, 24 and 48 h of continuous exposure.

b Combination Index (CI) values were calculated as mean values from three separate experiments, at fraction affected (FA) of 0.50, 0.75 and 0.90 in cells exposed to gemcitabine and pemetrexed combinations, as explained in the Materials and methods section.

**Table 2:** Cell cycle modulation after drug treatments, followed by 24-h culture in drug-free medium. Values (%) are means from three independent experiments and the differences (Δ) are calculated with respect to controls

**Cell lines**	**Treatment**	**G1 phase**	**(ΔG1)**	**S-phase**	**Δ(S)**	**G2/M phase**	**Δ(G2/M)**
	Control	70.50		27.16		2.34	
	Gemcitabine (1 h)	49.20	**−21.30**	46.57	**+19.41**	4.32	**+1.98**
*T24*	Pemetrexed (24 h)	57.05	**−13.35**	39.28	**+12.12**	3.67	**+1.33**
	Gemcitabine-pemetrexed	72.95	**+2.45**	23.76	**−3.4**	3.29	**+0.95**
	Pemetrexed-Gemcitabine	74.49	**+3.99**	16.80	**−10.36**	8.71	**+6.37**
							
	Control	44.32		39.46		16.22	
	Gemcitabine (1 h)	57.94	**+13.62**	33.46	**−6.00**	8.60	**−7.62**
*J82*	Pemetrexed (24 h)	37.95	**−6.37**	51.34	**+11.88**	10.71	**−5.51**
	Gemcitabine-pemetrexed	28.90	**−15.42**	61.20	**+21.74**	9.90	**−6.32**
	Pemetrexed-Gemcitabine	24.49	**−19.83**	61.12	**+21.66**	14.38	**−1.84**

**Table 3:** Effects of modulation of dCK and hENT on gemcitabine (dFdC, 1 h) and gemcitabine-pemetrexed (MTA) combinations IC_50_s (nM) in T24 and J82 cells. Values are means±s.d. of at least three separate experiments

**Cell lines**	**Treatments**	**IC_50_**	**+Deoxycytidine**	**+Dipyridamole**	**+NBMPR**
	dFdC	91.7±5.1	157.7±23.7	4513.4±909.5	7475.3±1337.3
	T24	dFdC-MTA	47.3±5.0	72.3±13.7	78.0±87.3	67.7±9.7
	MTA-dFdC	10.3±2.0	12.6±8.7	49.1±6.2	48.7±6.5	
	dFdC	4366.7±833.3	5863.0±234.5	4959.7±109.1	4476.5±801.3	
J82	dFdC-MTA	83.3±12.0	108.1±7.7	86.7±11.8	85.7±4.3	
	MTA-dFdC	100.0±9.7	111.1±12.3	101.3±2.7	99.7±5.2	

**Table 4:**
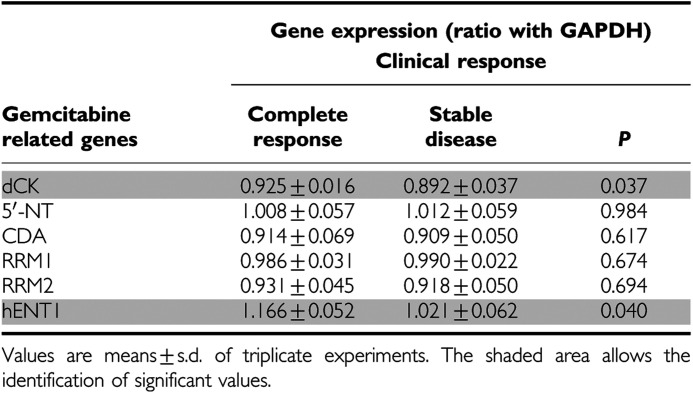
Correlation between clinical response and gene expression levels in treated patients
